# Absolute $\varphi- \vert C, \alpha, \beta; \delta\vert _{k}$ summability of infinite series

**DOI:** 10.1186/s13660-017-1445-5

**Published:** 2017-07-17

**Authors:** Smita Sonker, Alka Munjal

**Affiliations:** Department of Mathematics, National Institute of Technology Kurukshetra, Kurukshetra, 136119 India

**Keywords:** 40F05, 40D15, 40G05, absolute summability, infinite series, $\varphi- \vert C, \alpha, \beta; \delta \vert _{k}$ summability, quasi-*f*-power increasing sequence

## Abstract

In this paper, we established a generalized theorem on a minimal set of sufficient conditions for absolute summability factors by applying a sequence of a wider class (quasi-power increasing sequence) and the absolute Cesàro $\varphi-\vert C, \alpha, \beta; \delta \vert _{k}$ summability for an infinite series. We further obtained well-known applications of the above theorem as corollaries, under suitable conditions.

## Introduction

Let $\sum_{n=0}^{\infty }a_{n}$ be an infinite series with sequence of partial sums $\lbrace {s_{n}}\rbrace $ and the *n*th sequence to sequence transformation (mean) of $\lbrace {s_{n}}\rbrace $ be given by $u_{n}$ s.t.

1 $$ u_{n}=\sum_{k=0}^{\infty }u_{nk}s_{k}. $$

Before discussing $\varphi - \vert C, \alpha, \beta; \delta \vert _{k}$ summability, let us introduce some well-known basic summabilities which are helpful in understanding the $\varphi - \vert C, \alpha, \beta;\delta \vert _{k}$ summability.

### Definition 1

The series $\sum_{n=0}^{\infty }a _{n}$ is said to be absolute summable, if

2 $$ \lim_{n \rightarrow \infty }u_{n}=s $$

and $\sum_{n=1}^{\infty }\vert u_{n}-u_{n-1}\vert <\infty$.

### Definition 2

[1]

Let $t_{n}$ represent the *n*th $(C, 1)$ means of the sequence $(na_{n})$, then the series $\sum_{n=0}^{\infty }a_{n}$ is said to be $\vert C, 1\vert _{k}$ summable for $k\geq 1$, if

3 $$ \sum_{n=1}^{\infty }\frac{1}{n}\vert t_{n}\vert ^{k}< \infty. $$

### Definition 3

[2]

The *n*th Cesáro means of order $(\alpha, \beta)$, with $\alpha +\beta >-1$, of the sequence $(n a_{n}) $ is denoted by $t_{n}^{\alpha, \beta }$, *i.e.*

4 $$ t_{n}^{\alpha, \beta }=\frac{1}{A_{n}^{\alpha +\beta }}\sum _{v=1}^{n} A_{n-v}^{\alpha -1}A_{v}^{\beta }va_{v}, $$

where

$$A_{n}^{\alpha +\beta }= \textstyle\begin{cases} 0,&n< 0, \\ 1,&n=0, \\ O(n^{\alpha +\beta }),&n>0. \end{cases} $$

If the sequence $\lbrace t_{n}^{\alpha, \beta } \rbrace $ satisfies

5 $$ \sum_{n=1}^{\infty }\frac{\varphi_{n}^{k-1}}{n^{k}}\bigl\vert t_{n}^{\alpha, \beta }\bigr\vert ^{k}< \infty, $$

then the series $\sum_{n=0}^{\infty }a_{n}$ is said to be $\varphi -\vert C, \alpha, \beta \vert _{k}$ summable.

### Definition 4

For the following condition:

6 $$ \sum_{n=1}^{\infty }\frac{\varphi_{n}^{k-1}}{n^{k-\delta k}}\bigl\vert t_{n}^{\alpha, \beta }\bigr\vert ^{k}< \infty, $$

the series $\sum_{n=0}^{\infty }a_{n}$ is said to be $\varphi -\vert C, \alpha, \beta; \delta \vert _{k}$ summable, where $k\geq 1$, $\delta \geq 0$ and $(\varphi_{n})$ is a sequence of positive real numbers.

Bor gave a number of theorems on absolute summability. In 2002, Bor found the sufficient conditions for an infinite series to be $\vert C, \alpha \vert _{k}$ summable [3] and $\vert C, \alpha;\delta \vert _{k}$ summable [4]. In 2011, he generalized his previous results for $\vert C, \alpha, \beta \vert _{k}$ summability [5] and $\vert C, \alpha, \beta; \delta \vert _{k}$ summability [6], respectively. In 2014, Bor [7] generalized the $\vert C, \alpha \vert _{k}$ summability factor to the $\vert C, \alpha, \beta;\delta \vert _{k}$ summability of an infinite series and in [8], he discussed a general class of power increasing sequences and absolute Riesz summability factors of an infinite series. In [9], Bor applied $\vert C, \alpha, \gamma; \beta \vert _{k} $ summability to obtain the sufficient conditions for an infinite series to be absolute summable.

Bor [10] gave a new application of quasi-power increasing sequence by applying absolute Cesáro $\varphi -\vert C, \alpha \vert _{k}$ summability for an infinity series. Özarslan [11] generalized the result on $\varphi -\vert C, 1\vert _{k} $ by a more general absolute $\varphi -\vert C, \alpha \vert _{k}$ summability. In 2016, Sonker and Munjal [12] determined a theorem on generalized absolute Cesáro summability with the sufficient conditions for an infinite series and in [13], they used the concept of triangle matrices for obtaining the minimal set of sufficient conditions of an infinite series to be bounded.

## Known results

By using $\vert C, \alpha \vert _{k}$ summability, Bor [14] gave a minimal set of sufficient conditions for an infinite series to be absolute summable.

### Theorem 2.1

*Let* $X_{n}$ *be a quasi*-*f*-*power increasing sequence for some* *η* ($0<\eta <1 $). *Suppose also that there exists a sequence of numbers* $(A_{n}) $ *such that it is* *ξ*-*quasi*-*monotone satisfying the following*:

7 $$\begin{aligned}& \sum n \xi_{n} X_{n}=O(1), \end{aligned}$$

8 $$\begin{aligned}& \Delta A_{n} \leq \xi_{n}, \end{aligned}$$

9 $$\begin{aligned}& \vert \Delta \lambda_{n}\vert \leq \vert A_{n}\vert , \end{aligned}$$

10 $$\begin{aligned}& \sum A_{n} X_{n}\quad \textit{is convergent for all }n. \end{aligned}$$

*If the conditions*

11 $$\begin{aligned}& \vert \lambda_{n}\vert X_{n}=O(1)\quad \textit{as }n \rightarrow \infty, \end{aligned}$$

12 $$\begin{aligned}& \sum_{n=1}^{m} \frac{(w_{n}^{\alpha })^{k}}{n}=O(X_{m})\quad \textit{as }m\rightarrow \infty, \end{aligned}$$

*are satisfied*, *then the series* ${\sum a_{n}\lambda_{n}} $ *is* $\vert C, \alpha \vert _{k}$ *summable*, $0<\alpha \leq 1$ *and* $k \geq 1$.

## Main results

A positive sequence $X = (X_{n})$ is said to be a quasi-*f*-power increasing sequence if there exists a constant $K = K(X, f) \geq 1$ such that $K f_{n} X_{n} \geq f_{m} X_{m}$ for all $n \geq m \geq 1$, where $f = [f_{n}(\eta, \zeta)] = \lbrace n^{\eta }(\log n)^{\zeta },\zeta \geq 0,0 < \eta < 1\rbrace $ [15]. If we set $\zeta =0$, then we get a quasi-*η*-power increasing sequence [16].

With the help of generalized Cesáro $\varphi -\vert C, \alpha, \beta; \delta \vert _{k}$ summability, we modernized the results of Bor [14] and established the following theorem.

### Theorem 3.1

*Let* $X_{n}$ *be a quasi*-*f*-*power increasing sequence for some* *η* ($0<\eta <1$). *Suppose also that there exists a* *ξ*-*quasi*-*monotone sequence of numbers* $(A_{n}) $ *such that*

13 $$\begin{aligned}& \sum n \xi_{n} X_{n}=O(1), \end{aligned}$$

14 $$\begin{aligned}& \Delta A_{n} \leq \xi_{n}, \end{aligned}$$

15 $$\begin{aligned}& \vert \Delta \lambda_{n}\vert \leq \vert A_{n}\vert ,\quad \textit{and} \end{aligned}$$

16 $$\begin{aligned}& \sum A_{n} X_{n}\quad \textit{is convergent for all }n. \end{aligned}$$

*Then the series* ${\sum a_{n}\lambda_{n}} $ *is* $\varphi -\vert C, \alpha,\beta; \delta \vert _{k}$ *summable for* $k\geq 1$, $0<\alpha \leq 1$, $\beta > -1$, $\alpha +\beta >0 $ *and* $\delta \geq 0$, *if the following conditions are satisfied*:

17 $$\begin{aligned}& \vert \lambda_{n}\vert X_{n}=O(1)\quad \textit{as }n \rightarrow \infty, \end{aligned}$$

18 $$\begin{aligned}& \sum_{n=v}^{m} \frac{\varphi_{n}^{k-1}}{n^{(\alpha +\beta -\delta +1)k}}=O \biggl(\frac{ \varphi_{v}^{k-1}}{v^{(\alpha +\beta -\delta +1)k-1}} \biggr), \end{aligned}$$

19 $$\begin{aligned}& \sum_{n=1}^{m} \frac{\varphi_{n}^{k-1}(w_{n}^{\alpha, \beta })^{k}}{n ^{k-\delta k}}=O(X_{m})\quad \textit{as }m\rightarrow \infty, \end{aligned}$$

*where* $w_{n}^{\alpha, \beta } $ *is given by* [17]

20 $$ w_{n}^{\alpha, \beta }= \textstyle\begin{cases} \max_{1\leq v\leq n}\vert t_{v}^{\alpha, \beta }\vert , & \beta > -1, 0< \alpha < 1, \\ \vert t_{n}^{\alpha, \beta }\vert , & \beta > -1, \alpha =1. \end{cases} $$

## Lemmas

We need the following lemmas for the proof of our theorem.

### Lemma 4.1

[18]

*If* $0<\alpha \leq 1$, $\beta > -1 $ *and* $1\leq v\leq n$, *then*

21 $$ \Biggl\vert \sum_{p=0}^{v} A_{n-p}^{\alpha -1}A_{p}^{\beta }a_{p} \Biggr\vert \leq \max_{1\leq m\leq v} \Biggl\vert \sum _{p=0}^{m} A_{m-p}^{\alpha -1}A_{p}^{\beta }a_{p} \Biggr\vert . $$

### Lemma 4.2

[19]

*Let* $(X_{n}) $ *be a quasi*-*f*-*power increasing sequence for some* *η* ($0<\eta <1$). *If* $(A_{n}) $ *is a* *ξ*-*quasi*-*monotone sequence with* $\Delta A_{n} \leq \xi_{n} $ *and* $\sum n \xi_{n} X_{n} <\infty$, *then*

22 $$\begin{aligned}& \sum_{n=1}^{\infty }n X_{n}\vert A_{n}\vert < \infty, \end{aligned}$$

23 $$\begin{aligned}& nA_{n}X_{n}=O(1)\quad \textit{as }n\rightarrow \infty. \end{aligned}$$

## Proof of the theorem

Let $t_{n}^{\alpha, \beta }$ be the *n*th $(C, \alpha, \beta)$ mean of the sequence $(n a_{n} \lambda_{n} )$. Then the series will be $\varphi -\vert C, \alpha, \beta; \delta \vert _{k}$ summable (by Definition 4), if

24 $$ \sum_{n=1}^{\infty } \frac{\varphi_{n}^{k-1}}{n^{k-\delta k}}\bigl\vert T_{n}^{\alpha, \beta }\bigr\vert ^{k}< \infty. $$

Applying Abel’s transformation and Lemma 4.1, we have

25 $$\begin{aligned}& \begin{aligned}[b] T_{n}^{\alpha, \beta } &=\frac{1}{A_{n}^{\alpha +\beta }}\sum _{v=1}^{n} {A_{n-v}^{\alpha -1}A_{v}^{\beta }va_{v} \lambda _{v}} \\ &=\frac{1}{A_{n}^{\alpha +\beta }}\sum_{v=1}^{n-1} \Delta \lambda_{v} \sum_{p=1}^{v}{A_{n-p}^{\alpha -1}A_{p}^{\beta }pa _{p} }+\frac{\lambda_{n}}{A_{n}^{\alpha +\beta }}\sum_{v=1} ^{n}{A_{n-v}^{\alpha -1}A_{v}^{\beta }va_{v}} , \end{aligned} \end{aligned}$$

26 $$\begin{aligned}& \begin{aligned}[b] \bigl\vert T_{n}^{\alpha, \beta }\bigr\vert &\leq \frac{1}{A_{n}^{\alpha +\beta }} \sum_{v=1}^{n-1} \vert \Delta \lambda_{v}\vert \Biggl\vert \sum _{p=1}^{v}{A_{n-p}^{\alpha -1}A_{p}^{\beta }pa_{p} \Biggr\vert }+ \frac{\vert \lambda_{n}\vert }{A_{n}^{\alpha +\beta }} \Biggl\vert \sum _{v=1}^{n}{A_{n-v}^{\alpha -1}A_{v}^{\beta }va_{v}} \Biggr\vert \\ &\leq \frac{1}{A_{n}^{\alpha +\beta }}\sum_{v=1}^{n-1} {A_{v} ^{\alpha +\beta } w_{v}^{\alpha, \beta } \vert \Delta \lambda_{v}\vert }+ \vert \lambda_{n}\vert w_{n}^{\alpha, \beta } \\ &=T_{n,1}^{\alpha, \beta }+T_{n,2}^{\alpha, \beta }. \end{aligned} \end{aligned}$$

We use Minkowski’s inequality,

27 $$ \bigl\vert T_{n}^{\alpha, \beta }\bigr\vert ^{k} =\bigl\vert T_{n,1}^{\alpha, \beta }+T_{n,2}^{\alpha, \beta } \bigr\vert ^{k}\leq 2^{k} \bigl(\bigl\vert T_{n,1}^{\alpha, \beta }\bigr\vert ^{k}+\bigl\vert T_{n,2}^{\alpha, \beta }\bigr\vert ^{k} \bigr). $$

In order to complete the proof of the theorem, it is sufficient to show that

28 $$ \sum_{n=1}^{\infty } \frac{\varphi_{n}^{k-1}}{n^{k-\delta k}}\bigl\vert T_{n,r}^{\alpha, \beta }\bigr\vert ^{k}< \infty,\quad \text{for } r=1,2. $$

By using Hölder’s inequality, Abel’s transformation and the conditions of Lemma 4.2 [19], we have

29 $$\begin{aligned}& \begin{aligned}[b] \sum_{n=2}^{m+1} \frac{\varphi_{n}^{k-1}}{n^{k-\delta k}}\bigl\vert T_{n,1}^{\alpha, \beta }\bigr\vert ^{k} \leq {}&\sum _{n=2}^{m+1} \frac{ \varphi_{n}^{k-1}}{n^{k-\delta k}} \frac{1}{(A_{n}^{\alpha +\beta })^{k}} \Biggl(\sum_{v=1}^{n-1} {A_{v}^{\alpha +\beta } w_{v}^{\alpha, \beta } \vert \Delta \lambda_{v}\vert } \Biggr)^{k} \\ \leq {}&\sum_{n=2}^{m+1} \frac{\varphi_{n}^{k-1}}{n^{(1+\alpha +\beta -\delta) k}} \sum_{v=1}^{n-1} {v^{(\alpha +\beta)k} \bigl(w _{v}^{\alpha, \beta }\bigr)^{k} \vert A_{v}\vert } \Biggl(\sum_{v=1}^{n-1}\vert A_{v}\vert \Biggr)^{k-1} \\ ={}&O(1)\sum_{v=1}^{m} {v^{(\alpha +\beta)k} \bigl(w_{v}^{\alpha, \beta }\bigr)^{k} \vert A_{v} \vert }\sum_{n=v+1}^{m+1}\frac{\varphi_{n} ^{k-1}}{n^{(1+\alpha +\beta -\delta) k}} \\ ={}&O(1)\sum_{v=1}^{m} {v^{(\alpha +\beta)k} \bigl(w_{v}^{\alpha, \beta }\bigr)^{k} \vert A_{v} \vert }\frac{\varphi_{v}^{k-1}}{v^{(1+\alpha + \beta -\delta) k-1}} \\ ={}& O(1)\sum_{v=1}^{m} v \vert A_{v}\vert \bigl(w_{v}^{\alpha, \beta }\bigr)^{k} \frac{\varphi_{v}^{k-1}}{v^{k-\delta k}} \\ ={}& O(1)\sum_{v=1}^{m-1}\Delta \bigl(v \vert A_{v}\vert \bigr) \sum_{r=1} ^{v} \bigl(w_{r}^{\alpha, \beta }\bigr)^{k} \frac{\varphi_{r}^{k-1}}{r^{k- \delta k}} \\ & {} +O(1)m\vert A_{m}\vert \sum_{v=1}^{m} \bigl(w_{v}^{\alpha, \beta }\bigr)^{k} \frac{\varphi_{v}^{k-1}}{v^{k-\delta k}} \\ ={}& O(1)\sum_{v=1}^{m-1} \bigl\vert (v+1) \Delta \vert A_{v}\vert -\vert A_{v}\vert \bigr\vert X_{v}+O(1) m\vert A_{m}\vert X_{m} \\ ={}& O(1)\sum_{v=1}^{m-1}v \vert \Delta A_{v}\vert X_{v}+O(1)\sum_{v=1}^{m-1} \vert A_{v}\vert X_{v}+O(1) m\vert A_{m} \vert X_{m} \\ ={}& O(1)\sum_{v=1}^{m-1}v \xi_{v} X_{v}+O(1)\sum_{v=1} ^{m-1}\vert A_{v}\vert X_{v}+O(1) m\vert A_{m}\vert X_{m} \\ ={}&O(1)\quad \text{as } m\rightarrow \infty, \end{aligned} \end{aligned}$$

30 $$\begin{aligned}& \begin{aligned}[b] \sum_{n=2}^{m} \frac{\varphi_{n}^{k-1}}{n^{k-\delta k}}\bigl\vert T_{n,2}^{\alpha, \beta }\bigr\vert ^{k} ={}&O(1)\sum_{n=1}^{m} \vert \lambda_{n}\vert \bigl(w _{n}^{\alpha, \beta } \bigr)^{k} \frac{\varphi_{n}^{k-1}}{n^{k-\delta k}} \\ ={}&O(1)\sum_{n=1}^{m-1}\Delta \vert \lambda_{n}\vert \sum_{v=1} ^{n} \bigl(w_{v}^{\alpha, \beta }\bigr)^{k} \frac{\varphi_{v}^{k-1}}{v^{k- \delta k}} +O(1)\vert \lambda_{m}\vert \sum_{n=1}^{m} \bigl(w_{n}^{\alpha, \beta }\bigr)^{k} \frac{\varphi_{n}^{k-1}}{n^{k-\delta k}} \\ ={}&O(1)\sum_{n=1}^{m-1} \vert \Delta \lambda_{n}\vert X_{n}+O(1)\vert \lambda_{m} \vert X_{m} \\ ={}&O(1)\sum_{n=1}^{m-1} \vert A_{n}\vert X_{n}+O(1)\vert \lambda_{m}\vert X_{m} \\ ={}&O(1) \quad \text{as }m\rightarrow \infty. \end{aligned} \end{aligned}$$

Collecting (24)-(30), we have

31 $$ \sum_{n=1}^{\infty }\frac{\varphi_{n}^{k-1}}{n^{k-\delta k}}\bigl\vert T_{n}^{\alpha, \beta }\bigr\vert ^{k}< \infty. $$

Hence the proof of the theorem is completed.

## Corollaries

### Corollary 6.1

*Let* $X_{n}$ *be a quasi*-*f*-*power increasing sequence for some* *η* ($0<\eta <1$) *and there exists a sequence of numbers* $(A_{n}) $ *such that it is* *ξ*-*quasi*-*monotone satisfying* (13)-(17) *and the following condition*:

32 $$ \sum_{n=1}^{m} \frac{{(w_{n}^{\alpha, \beta })^{k}}}{n^{1-\delta k}}=O(X_{m})\quad \textit{as }m\rightarrow \infty, $$

*then the series* ${\sum a_{n}\lambda_{n}} $ *is* $\vert C, \alpha, \beta;\delta \vert _{k} $ *summable*, $\alpha +\beta >\delta$, $0<\alpha \leq 1$, $\beta >-1$, $\delta \geq 0$, $k\geq 1$, *where* $w_{n}^{ \alpha, \beta } $ *is given by* (20).

### Proof

On putting $\varphi_{n}=n $ in Theorem 3.1, we will get (32) and the following condition:

33 $$ \sum_{n=v}^{m} \frac{1}{n^{1+k(\alpha +\beta -\delta)}}=O \biggl(\frac{1}{v ^{(\alpha +\beta -\delta)k}} \biggr). $$

Here, condition (33) always holds. We omit the details as the proof is similar to that of Theorem 3.1 using the conditions (33) and (32) instead of (18) and (19). □

### Corollary 6.2

*Let* $X_{n}$ *be a quasi*-*f*-*power increasing sequence for some* *η* ($0<\eta <1 $) *and there exists a sequence of numbers* $(A_{n}) $ *such that it is* *ξ*-*quasi*-*monotone satisfying* (13)-(17) *and the following conditions*:

34 $$\begin{aligned}& \sum_{n=v}^{m} \frac{\varphi_{n}^{k-1}}{n^{k(1+\alpha +\beta)}}=\frac{ \varphi_{v}^{k-1}}{v^{k(1+\alpha +\beta)-1}}, \end{aligned}$$

35 $$\begin{aligned}& \sum_{n=1}^{m} \frac{{(w_{n}^{\alpha, \beta })^{k}}\varphi_{n}^{k-1}}{n ^{k}}=O(X_{m})\quad \textit{as }m\rightarrow \infty, \end{aligned}$$

*then the series* ${\sum a_{n}\lambda_{n}} $ *is* $\varphi -\vert C, \alpha,\beta \vert _{k}$ *summable*, $\alpha +\beta >0$, $0<\alpha \leq 1$, $\beta > -1$, $k\geq 1$, *where* $w_{n}^{\alpha, \beta } $ *is given by* (20).

### Proof

On putting $\delta =0 $ in Theorem 3.1, we will get (34) and (35). We omit the details as the proof is similar to that of Theorem 3.1 using the conditions (34) and (35) instead of (18) and (19). □

### Corollary 6.3

[14]

*Let* $X_{n}$ *be a quasi*-*f*-*power increasing sequence for some* *η* ($0<\eta <1$) *and there exists a sequence of numbers* $(A_{n}) $ *such that it is* *ξ*-*quasi*-*monotone satisfying* (13)-(17) *and the following conditions*:

36 $$ \sum_{n=1}^{m} \frac{{(w_{n}^{\alpha })^{k}}}{n}=O(X_{m}) \quad \textit{as }m\rightarrow \infty, $$

*then the series* ${\sum a_{n}\lambda_{n}} $ *is* $\vert C, \alpha \vert _{k}$ *summable*, $0<\alpha \leq 1$, $k\geq 1$, *where* $w_{n}^{\alpha } $ *is given by*

37 $$ w_{n}^{\alpha }= \textstyle\begin{cases} \vert t_{n}^{\alpha }\vert ,& \alpha =1, \\ \max_{1\leq v\leq n}\vert t_{v}^{\alpha }\vert ,& 0< \alpha < 1. \end{cases} $$

### Proof

On putting $\varphi_{n}=n $, $\delta =0 $ and $\beta =0 $ in Theorem 3.1, we will get (36) and the following condition:

38 $$ \sum_{n=v}^{m} \frac{1}{n^{1+k\alpha }}=O \biggl(\frac{1}{v^{k\alpha }} \biggr). $$

Here, condition (38) always holds. We omit the details as the proof is similar to that of Theorem 3.1 using the conditions (38) and (36) instead of (18) and (19). □

## Conclusion

The aim of our paper is to obtain the minimal set of sufficient conditions for an infinite series to be absolute Cesáro $\varphi -\vert C, \alpha, \beta; \delta \vert _{k}$ summable. Through the investigation, we may conclude that our theorem is a generalized version which can be reduced for several well-known summabilities as shown in the corollaries. Further, our theorem has been validated through Corollary 6.3, which is a result of Bor [14].
